# VAMP7‐mediated autophagy regulates cervical cancer progression associated with persistent HPV16 infection

**DOI:** 10.1002/ctm2.70590

**Published:** 2026-01-08

**Authors:** Weijuan Xin, Junjie Zhang, Dan Wu, Yiying Cai, Xue Ding, Lu Zhou, Na Liu, Yue Wang, Zhiling Zhu, Keqin Hua

**Affiliations:** ^1^ Department of Obstetrics and Gynecology Obstetrics and Gynecology Hospital of Fudan University Shanghai China; ^2^ Shanghai Key Lab of Reproduction and Development Shanghai China; ^3^ Shanghai Key Lab of Female Reproductive Endocrine Related Diseases Shanghai China; ^4^ Department of Gastroenterology Tongji Hospital, Tongji University School of Medicine Shanghai China; ^5^ Affiliated Hospital of Shandong Second Medical University Weifang China; ^6^ Department of Pathology People's Hospital of Dezhou Dezhou China

**Keywords:** autophagy, cervical cancer, HPV16 infection, VAMP7, SNARE

## Abstract

**Backgroud:**

Persistent infection with high‐risk human papillomavirus type 16 (HPV16) is a principal etiological factor in cervical cancer. Nevertheless, the molecular events linking HPV16‐associated lesion progression to malignant transformation remain insufficiently characterized, particularly those involving vesicular trafficking and autophagy regulation.

**Methods:**

Proteomic analysis was conducted across five stages of HPV16‐associated cervical lesion progression to identify differentially expressed proteins. The expression of vesicle‐associated membrane protein 7 (VAMP7) was validated in cervical tissue specimens and cellular models. Gain‐ and loss‐of‐function approaches were employed to assess the effects of VAMP7 on cellular proliferation, migration, invasion, and apoptosis. Autophagic activity was evaluated by LC3 lipidation, autophagosome accumulation, and analysis of SNARE complexrelated proteins. The in vivo effects of VAMP7 were examined using xenograft tumor models.

**Results:**

VAMP7 demonstrated dynamic expression changes during cervical lesion progression, characterized by decreased expression in HPV16‐positive non‐malignant tissues and a gradual increase with disease severity, reaching the highest levels in advanced cervical cancer. Functionally, VAMP7 enhanced proliferation, migration, and invasion while inhibiting apoptosis in cervical cancer cells, whereas distinct effects were observed in non‐tumor cervical epithelial cells. Mechanistically, VAMP7 regulated autophagic flux through modulation of SNARE‐mediated vesicle fusion, resulting in altered autophagosome accumulation and autophagy‐related signaling. In xenograft models, VAMP7 overexpression significantly promoted tumor growth and increased the expression of autophagy‐associated markers.

**Conclusion:**

These data indicate that dysregulation of VAMP7‐mediated autophagy contributes to cervical carcinogenesis in an HPV16‐associated context. VAMP7 may represent a potential therapeutic target for the treatment of cervical cancer.

**Key points:**

- VAMP7 displays dynamic expression changes during HPV16‐associated cervical lesion progression.
- VAMP7 promotes malignant phenotypes of cervical cancer cells by regulating autophagic flux via SNARE‐mediated vesicle fusion.
- Dysregulated VAMP7‐mediated autophagy contributes to cervical carcinogenesis in an HPV16‐associated context.

## Graphical abstract

Vesicle‐associated membrane protein 7 (VAMP7) plays a pivotal role in HPV16‐driven cervical cancer by regulating autophagy, promoting tumour cell survival, immune evasion and lesion progression. These findings highlight VAMP7 as a potential therapeutic target, offering new opportunities for early intervention and treatment of cervical cancer.

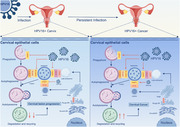

## 1 INTRODUCTION

Cervical cancer (CC) is the fourth most prevalent cancer in women and is predominantly caused by persistent infection with high‐risk human papillomavirus (HR‐HPV), especially types 16 and 18, with HPV16 being the most virulent.^1^ Although most HPV infections resolve within 2 years, a minority persist, leading to chronic damage and abnormal proliferation of cervical epithelial cells.^2^ These infected cells gradually transform from normal to low‐grade squamous intraepithelial lesions (LSIL), then to high‐grade squamous intraepithelial lesions (HSIL), and eventually to CC.^2^ The persistence and progression of HPV16 infection to cervical lesions and ultimately to malignancy are influenced by a multitude of cellular pathways and molecular mechanisms.^3^, ^4^, ^5^

Autophagy, a fundamental cellular process for degrading and recycling cellular components via lysosomal fusion, is crucial for maintaining homeostasis.^6^ It plays a vital role in immune responses and tumour suppression.^7^, ^8^ During viral infections, autophagy helps clear pathogens and regulate immune signalling, preventing viral replication.^9^ Additionally, autophagy inhibits early tumour development by removing damaged organelles and proteins.^7^ Soluble N‐ethylmaleimide‐sensitive factor attachment protein receptor (SNARE) proteins are essential for autophagy, mediating the fusion of autophagosomes with lysosomes.^10^ Proper SNARE function ensures effective autophagy, and any dysfunction can disrupt this process, leading to impaired cellular function and tumour formation. Thus, understanding SNARE proteins in autophagy is critical for developing therapeutic strategies against viral infections and cancer.

Proteomics, the large‐scale study of proteins, is a powerful tool for investigating these changes.^11^, ^12^ By analysing the entire protein complement of a cell or tissue, proteomics can provide comprehensive insights into cellular processes and disease mechanisms. Previous studies have utilised proteomics techniques such as two‐dimensional fluorescence difference gel electrophoresis (2D‐DIGE) and mass spectrometry (MALDI‐TOF) to identify differential protein expression in HPV‐negative and HPV‐positive normal cervical cells.^13^ For instance, Idanya et al. identified four proteins with increased expression and three with decreased expression in HPV‐infected cells. However, these studies did not delve into protein changes and molecular mechanisms during the progression of CC.

This study utilises proteomics techniques to perform label‐free quantitative screening of key proteins at different stages of HPV16 infection, identifying vesicle‐associated membrane protein 7 (VAMP7), a member of the SNARE family, as an important protein influencing CC progression. Our study is to elucidate the regulatory role of VAMP7 in the progression of HPV16‐infected cervical lesions through autophagy mediated by the SNARE vesicular transport pathway. By investigating the mechanistic interactions between VAMP7 and autophagic pathways in HPV16‐infected cervical cells, we seek to provide novel insights into the pathogenesis of CC and identify potential therapeutic targets for intervention.

## 2 RESULTS

### 2.1 Proteomic analysis reveals 519 protein alterations in cervical lesion progression induced by persistent HPV16 infection

**FIGURE 1:**
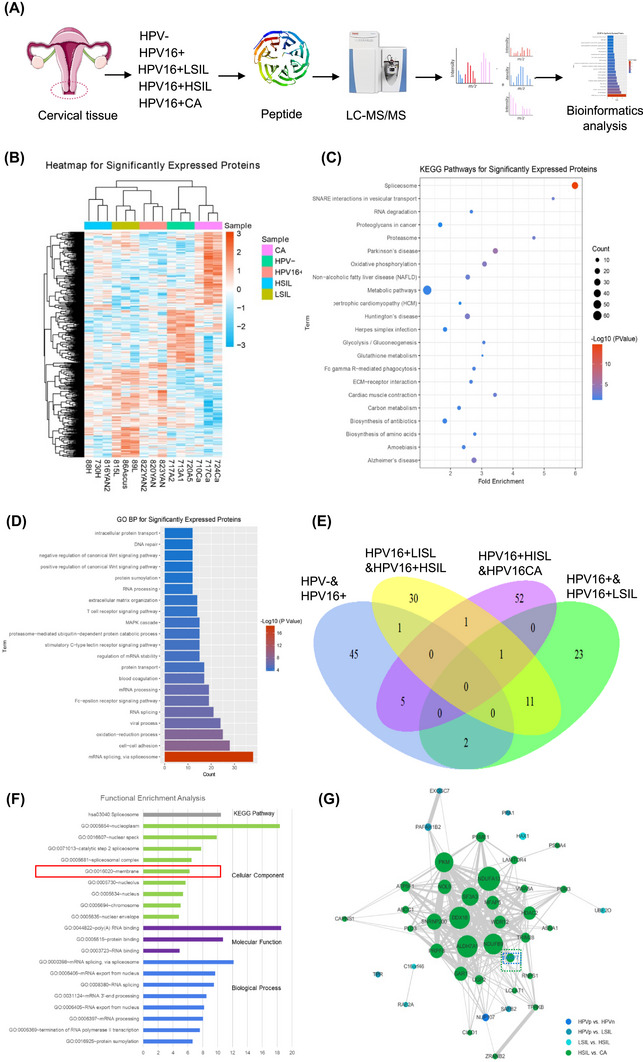
Proteomic analysis reveals protein alterations in cervical lesion progression induced by persistent HPV16 infection. (A) Workflow of label‐free quantitative (LFQ) proteomics analysis performed on tissue samples from five distinct stages of cervical lesion progression: HPV‐negative, HPV16+, HPV16+ LSIL, HPV16+ HSIL and HPV16+ CA. (B) Heatmap of hierarchical clustering of differentially expressed proteins across samples from the five stages, highlighting protein expression patterns that distinguish each stage. The colour scale represents normalised expression levels, with red indicating upregulation and blue indicating downregulation. (C) Kyoto encyclopedia of genes and genomes (KEGG) pathway enrichment analysis for differentially expressed proteins, providing insight into the signalling pathways most significantly associated with cervical lesion progression at different stages. (D) Gene Ontology (GO) biological process (GO‐BP) enrichment analysis for differentially expressed proteins across the five stages, identifying key biological processes enriched in each group. The top enriched terms are shown with corresponding *p* values. (E) Venn diagram illustrating pairwise comparisons of differentially expressed proteins between four groups derived from the five stages of cervical lesion progression. Each circle represents a comparison between two stages, with overlapping areas indicating shared differentially expressed proteins. The number of proteins shared or unique to each stage comparison is shown. (F) KEGG and GO‐Biological Process (GO‐BP) enrichment analyses of differentially expressed proteins identified in Module 1 of the Weighted Gene Co‐expression Network Analysis (WGCNA) network across the five pathological stages of cervical lesion progression. (G) Co‐expression submodule of Module 1 highlighting key protein nodes associated with disease progression. Node size represents degree (number of connections), node colour corresponds to comparison groups and edge thickness reflects correlation strength. Proteins with higher degree and centrality values are indicated as critical regulators within the module.

To investigate protein level changes in cervical tissues during persistent HPV16 infection, we performed label‐free quantitative proteomic analysis using liquid chromatography/mass spectrometry (LC/MS) on samples from five stages: HPV‐negative (HPV−), HPV16‐positive (HPV16+), HPV16‐positive and low‐grade intraepithelial neoplasia (HPV16+LSIL), HPV16‐positive and high‐grade intraepithelial neoplasia (HPV16+HSIL), and HPV16‐positive and cervical cancer (HPV16+CA). Each group included three biological replicates, resulting in a total of 15 samples (Table S1). All samples were processed with the FASP approach, and peptides were analysed via LC–MS/MS. Relative quantitation was achieved using MaxQuant and the UniProt database, with strict criteria (<1% false discovery rate [FDR] at both peptide and protein group levels; Figure 1A). Over 5000 proteins were identified per replicate, with 4368 non‐redundant proteins identified across groups.

To ensure the reliability of the proteomic data, we evaluated the consistency of the LC–MS/MS results across the 15 samples. Box plot analysis of label‐free quantitative (LFQ) intensities demonstrated consistent average intensity levels across all samples, confirming that there was no systemic bias in the quantification process (Figure S1A). Further, linear regression analysis revealed high correlation coefficients (around .9) between biological replicates within each group, indicating the high reproducibility of the data (Figure S1B). These results confirm that the observed variations in protein expression are reliable and robust.

To visualise global protein expression patterns across different pathological states, we generated a heatmap of differentially expressed proteins (Figure 1B). The heatmap revealed heterogeneous protein expression profiles among the five groups, reflecting substantial inter‐individual variability, particularly within HPV16‐positive lesion groups. These patterns highlight distinct molecular features associated with different pathological conditions. Notably, differences in protein expression were observed between HPV16+ LSIL and HPV16+ CA samples; however, variability within intermediate stages such as HPV16+ HSIL suggests that cervical lesion evolution is not uniform across individuals. The HPV‐negative group displayed a distinct expression profile compared with HPV‐positive lesions, underscoring differences between non‐infected and infected tissues. Unsupervised clustering highlighted heterogeneous protein expression patterns across pathological groups, facilitating the identification of proteins associated with lesion severity.

We performed Gene Ontology‐biological process (GO‐BP) and KEGG pathway enrichment analyses on significantly expressed proteins to identify key functional pathways associated with cervical lesion progression (Figure 1C,D). In the KEGG pathway analysis, several pathways related to RNA splicing were significantly enriched, indicating that RNA splicing may play a critical role in the pathogenesis and progression of CC. The GO analysis identified several enriched biological processes, including RNA splicing, viral processes, oxidation–reduction processes, cell–cell adhesion and mRNA splicing via the spliceosome. These enrichments suggest that the regulation of RNA processing, cellular adhesion and redox balance are important factors in the molecular mechanisms underlying HPV infection and its progression to malignancy.

### 2.2 VAMP7 in key protein modules linked to cervical lesion progression associated with persistent HPV16 infection

To better understand the changes in protein expression across different stages of cervical lesion progression, we analysed the differential protein expression in five distinct groups representing disease severity: HPV‐negative (HPV−), HPV16‐positive (HPV16+), HPV16‐positive with low‐grade squamous intraepithelial lesion (HPV16+LSIL), HPV16‐positive with high‐grade squamous intraepithelial lesion (HPV16+HSIL) and HPV16‐positive cervical cancer (HPV16+CA). Among these groups, we focused on key pairwise comparisons: HPV− versus HPV16+, HPV16+ versus HPV16+LSIL, HPV16+LSIL versus HPV16+HSIL, and HPV16+HSIL versus HPV16+CA, to identify proteins whose expression varied significantly during the progression of cervical lesions. The greatest number of differentially expressed proteins was observed between the HPV16+HSIL and HPV16+CA groups, with 59 proteins showing significant changes. This suggests that the transition from HSIL to invasive CC involves substantial alterations at the protein level (Figure 1E).

To further investigate the protein co‐expression patterns associated with cervical lesion progression induced by persistent HPV16 infection, we constructed a protein co‐expression network using Weighted Gene Co‐expression Network Analysis (WGCNA) based on MS data of over 4000 proteins. This analysis resulted in 13 distinct modules (Table 1). Among these, Module 1 exhibited a significant positive correlation with CC progression, indicating that the proteins within this module may play crucial roles in disease advancement. Module 1 consists of 730 proteins, of which 129 are differentially expressed across the five disease stages. The proteins in this module are significantly enriched in biological processes such as RNA splicing and protein ubiquitination, as revealed by GO terms and KEGG pathway analyses (Figure 1F). We further analysed the proteins within Module 1 that were differentially expressed at various stages of cervical lesion progression, focusing on the following comparisons: HPV− versus HPV16+, HPV16+ versus HPV16 + LSIL, HPV16 + LSIL versus HPV16 + HSIL, and HPV16 + HSIL versus HPV16 + CA. The comparison between HPV16 + HSIL and HPV16 + CA revealed the highest number of differentially expressed proteins (31 proteins), suggesting that the transition from HSIL to CC involves significant molecular changes. To explore the functional relevance of these proteins in CC progression, we extracted a co‐expression submodule from Module 1 (Figure 1G). This submodule consisted of proteins that were significantly differentially expressed in at least one of the four pairwise group comparisons. By calculating network parameters such as node degree, betweenness centrality and closeness centrality, we identified the top 30% of proteins with the highest network connectivity, which are likely key regulators in the progression of CC (Figure 1G).

**TABLE 1:** Protein co‐expression network modules associated with cervical lesion progression caused by HPV16 infection.

Modules	No. of proteins	No. of DE‐proteins	Correlation coefficient	*p* value
1	730	129	.62	.01
2	945	116	−.50	.06
3	381	42	−.50	.06
4	134	8	−.49	.06
5	66	4	.40	.14
6	124	15	.38	.16
7	254	31	−.35	.20
8	126	55	−.24	.39
9	175	1	.09	.76
10	302	78	−.09	.76
11	87	4	−.08	.79
12	78	1	.03	.91
13	336	3	−.01	.97

Among the proteins identified in the submodule, eight core proteins were highlighted for their significant roles in disease progression: PRPF8, SNRNP200, SF3A3, PYCRL, NOLA1, PKM2, VAMP8 and VAMP7. Notably, VAMP7 emerged as a key protein within the differentially expressed proteins, demonstrating significant expression changes across multiple disease stages. Given its prominent role, VAMP7 was identified as a core protein potentially playing an essential role in CC progression. Further validation of its function in CC development will provide deeper insights into its mechanistic involvement in the disease.

### 2.3 VAMP7 exhibits biphasic expression in HPV16‐driven cervical lesions and correlates with disease progression and aggressive tumour behaviour

**FIGURE 2:**
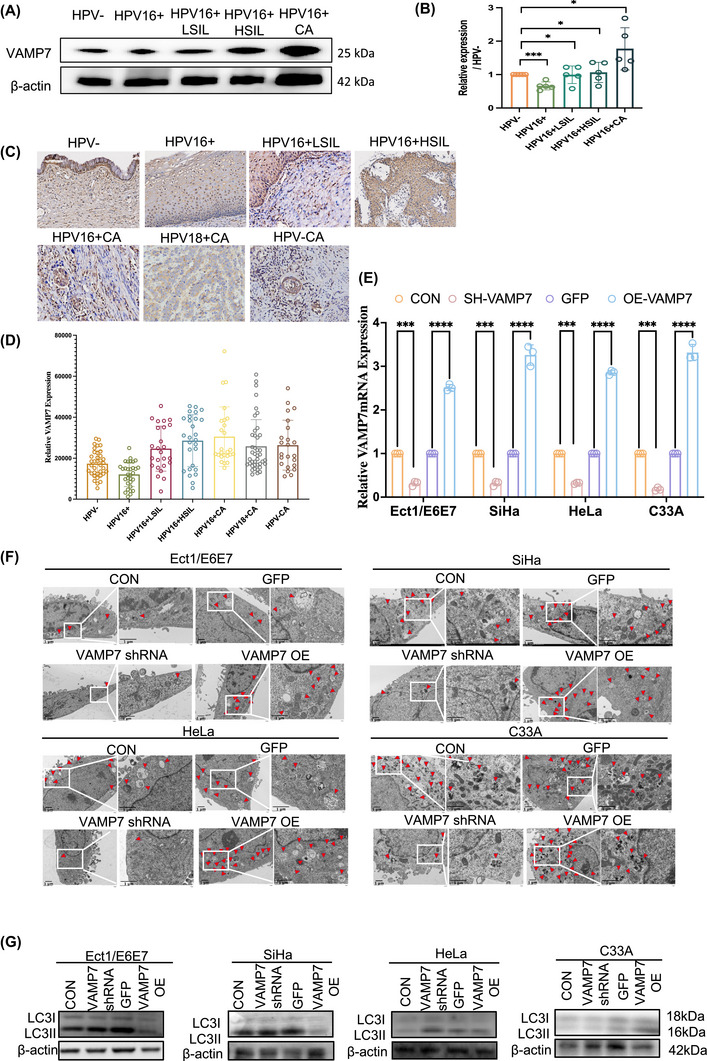
Vesicle‐associated membrane protein 7 (VAMP7) in key protein modules linked to cervical cancer induced by persistent HPV16 infection. (A, B) Expression profile of VAMP7 across five disease stages (HPV−, HPV16^+^, HPV16^+^ LSIL, HPV16^+^ HSIL, HPV16^+^ CA), demonstrating the dynamic changes in VAMP7 abundance during disease progression. Individual data points represent biological replicates (*n* = 5), and bars indicate mean ± standard deviation (SD). (C, D) Representative immunohistochemistry (IHC) staining images showing VAMP7 expression patterns across eight cervical tissue groups. Scale bars are indicated. Data are presented as mean ± SD with individual data points (*n* = 30). Statistical significance was determined using one‐way analysis of variance (ANOVA). *p < .05. (E) Quantification of VAMP7 protein expression normalised to β‐actin in Ect1/E6E7, SiHa, HeLa and C33A cells under the indicated conditions. Data are presented as relative expression levels compared with control groups. Statistical significance was determined by one‐way ANOVA with multiple comparisons. ***p < .001, ****p < .0001. (F) Representative transmission electron microscopy (TEM) images showing autophagosome formation in cervical epithelial and cancer cells following VAMP7 regulation. Quantification of autophagic vesicles per cell is shown with individual data points (*n* = 3). Scale bar = 1 µm. (G) Western blot analysis showing the effect of VAMP7 knockdown or overexpression on the autophagy marker LC3 (LC3‐I and LC3‐II) in Ect1/E6E7, SiHa, HeLa and C33A cell lines. Representative blots and quantitative analysis are shown.

Proteomic screening followed by bioinformatic filtering identified eight candidate proteins with differential expression across five groups of cervical tissues. Western blot validation confirmed that all proteins were upregulated in the HPV16^+^ CA group, except for PKM2, which was downregulated (Figures 2A,B and S1C). These statistically significant changes (*p* < .05) support the robustness of the proteomic findings and suggest their potential involvement in CC biology. Among these proteins, PYCRL, VAMP8 and PKM2 were significantly increased in HPV16^+^ tissues, whereas VAMP7 displayed a distinct biphasic pattern, being downregulated in the HPV16^+^ group but progressively increasing in HPV16^+^ LSIL, HPV16^+^ HSIL and HPV16^+^ CA. This dynamic expression trend implies that VAMP7 may participate in the progression from high‐grade lesions to carcinoma during HPV16‐driven cervical neoplasia.

To examine whether this pattern also occurs in other HPV backgrounds, immunohistochemistry (IHC) was performed on seven groups of cervical samples, including HPV−, HPV16^+^, HPV16^+^ LSIL, HPV16^+^ HSIL, HPV16^+^ CA, HPV18^+^ CA and HPV− CA (Table S2). Consistent with Western blotting results, VAMP7 expression in HPV16^+^ lesions initially decreased and then increased with disease severity, with the highest levels observed in HPV16^+^ CA (Figures 2C,D and S1D). Notably, VAMP7 expression was also elevated in HPV18^+^ CA and HPV− CA compared with HPV− normal cervical tissue. These findings indicate that VAMP7 upregulation is not restricted to HPV16 infection, and may also be associated with CC progression irrespective of HPV genotype.

To further explore the clinical significance of VAMP7 in CC, we analysed 68 CC samples, including 30 HPV16^+^, 22 HPV18^+^ and 16 HPV− cases (Table S2). High VAMP7 expression was significantly correlated with FIGO stage, lymphovascular invasion and recurrence (*p* < .05; Table 2), supporting its association with aggressive tumour behaviour.

**TABLE 2:** Association between vesicle‐associated membrane protein 7 (VAMP7) expression and clinicopathological characteristics of patients with cervical cancer (CC).

	VAMP7		
Characteristics	Low expression (*N*%)	High expression (*N*%)	*p*
Age (years)			.622
<45	13 (19.12)	15 (22.06)	
≥45	21 (30.88)	19 (27.94)	
Histological type			.223
Squamous cell carcinoma	26 (38.24)	20 (29.41)	
Adenocarcinoma	2 (2.94)	8 (11.77)	
Adenosquamous carcinoma	4 (5.88)	4 (5.88)	
Small cell neuroendocrine carcinoma	2 (2.94)	2 (2.94)	
FIGO stages			.048*
I	20 (29.41)	10 (14.71)	
II	6 (8.82)	9 (13.24)	
III	8 (11.76)	15 (22.06)	
Histological grades			.209
High differentiation	15 (22.06)	10 (14.71)	
Low/moderate differentiation	19 (27.94)	24 (35.29)	
Tumour diameters (cm)			.176
≤2	2 (2.94)	2 (2.94)	
>2, ≤4	25 (36.76)	18 (26.47)	
>4	7 (10.29)	14 (20.59)	
Myometrial invasion			.784
Inner 1/3	6 (8.82)	7 (10.29)	
Middle 1/3	1 (1.47)	2 (2.94)	
Outer 1/3	27 (39.71)	25 (36.77)	
Lymphovascular invasion			.049*
Present	16 (23.53)	24 (35.39)	
Absent	18 (26.47)	10 (14.71)	
Surgical margins			.555
Positive	2 (2.94)	1 (1.47)	
Negative	32 (47.06)	33 (48.53)	
Parametrial infiltration			.549
Present	6 (8.82)	8 (11.76)	
Absent	28 (41.18)	26 (38.24)	
Lower uterine segments involved			.770
Present	7 (10.29)	8 (11.76)	
Absent	27 (39.71)	26 (38.24)	
Lymph node metastasis			.073
Present	8 (11.76)	15 (22.06)	
Absent	26 (38.24)	19 (27.94)	
Other sites metastatic			
Present	0 (0)	3 (4.41)	.076
Absent	34 (50)	31 (45.59)	
Recrudesce			.040*
Present	2 (2.94)	8 (11.76)	
Absent	32 (47.06)	26 (38.24)	
HPV typing			.298
16+	17 (25)	13 (19.12)	
18+	8 (11.76)	14 (20.59)	
Negative	9 (13.24)	7 (10.29)	

* *p* < .05.

Together, these results suggest that although VAMP7 exhibits a characteristic ‘decrease‐then‐increase’ pattern during HPV16‐associated lesion progression, its overall upregulation in HPV18^+^ and HPV− CC indicates that VAMP7 may contribute broadly to CC development, potentially acting at the interface between HPV‐driven molecular alterations and tumour progression.

### 2.4 VAMP7 regulates autophagy and modulates disease progression in cervical lesions

VAMP7, a member of the SNARE protein family, plays a critical role in regulating membrane fusion processes, particularly in autophagy.^10^, ^14^ SNARE proteins are essential for the fusion of autophagosomes with lysosomes, a key step in the degradation and recycling of cellular components.^14^ VAMP7, in particular, is known to facilitate vesicular trafficking and is crucial for maintaining cellular homeostasis through autophagy.^15^ Given its involvement in autophagic processes, we hypothesised that VAMP7 may play a pivotal role in regulating autophagy and influencing key phenotypic changes in cervical cells during HPV infection.

To investigate this hypothesis, we analysed the effects of VAMP7 overexpression and knockdown in both HPV16‐positive and HPV‐negative cervical cell lines, including SiHa, HeLa, C33A and Ect1/E6E7 (Figures 2E and S2A). Our experiments demonstrated that VAMP7 overexpression and knockdown both led to significant changes in autophagic activity, as well as in cell proliferation, apoptosis, migration and invasion, suggesting that VAMP7 modulates these processes through its regulation of autophagy.

Electron microscopy results showed that VAMP7 knockdown significantly reduced the number of autophagosomes, while VAMP7 overexpression resulted in an increase in autophagosomes in cervical cells (Figure 2F). This suggests that VAMP7 is crucial for autophagy in cervical cells and that its overexpression enhances autophagic flux, while its knockdown inhibits the formation of autophagosomes. These findings strongly support the hypothesis that VAMP7 regulates autophagy in cervical cells.

To further validate these results, Western blot analysis was conducted to measure the levels of LC3B, a key marker of autophagy. VAMP7 overexpression significantly increased LC3B expression, while VAMP7 knockdown led to a marked reduction in LC3B levels (Figure 2G), further corroborating the role of VAMP7 in promoting autophagy. These results are consistent with the electron microscopy findings, demonstrating that VAMP7 regulates autophagy in cervical cells through its role in autophagosome formation and fusion.

**FIGURE 3:**
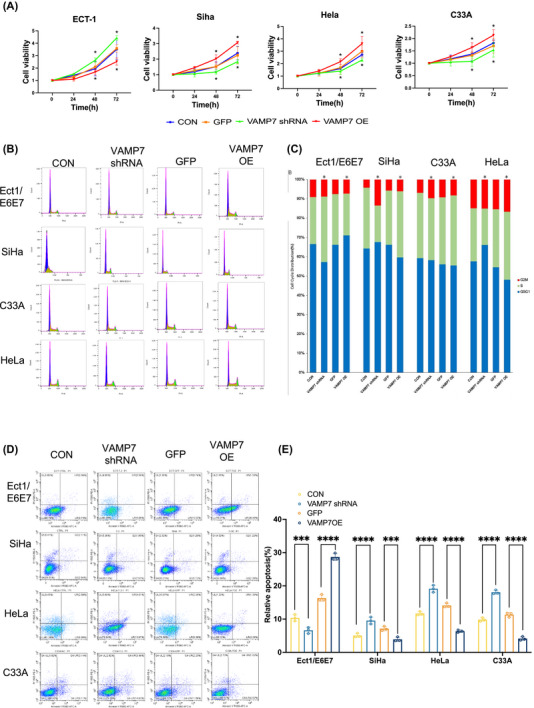
The effects of vesicle‐associated membrane protein 7 (VAMP7) regulation on cell proliferation, cell cycle and apoptosis in cervical cells. (A) Cell proliferation was assessed by CCK‐8 assay following VAMP7 knockdown or overexpression. Data are presented as mean ± standard deviation (SD) with individual data points representing independent biological replicates (*n* = 3). (B, C) Flow cytometry analysis assessing the effects of VAMP7 knockdown or overexpression on cell‐cycle distribution in cervical epithelial and cancer cells. (D, E) Flow cytometry analysis of apoptosis in cervical cancer cells after VAMP7 knockdown or overexpression. Representative plots and quantitative analysis are shown. Data are presented as mean ± SD from three independent experiments (*n* = 3). Statistical significance was determined using two‐way analysis of variance (ANOVA) with post hoc testing. *p < .05.

In addition to its role in autophagy, we also observed that VAMP7 regulation had a differential impact on cell proliferation, apoptosis and migration, which are key phenotypic changes associated with CC progression. VAMP7 overexpression promoted cell proliferation in SiHa, HeLa and C33A cells, while inhibiting proliferation in Ect1/E6E7 cells (Figure 3A–C). This suggests that VAMP7 differentially regulates cell proliferation in a context‐dependent manner, potentially through its impact on autophagy.

Flow cytometry analysis showed that alterations in VAMP7 expression were accompanied by changes in apoptotic profiles across cervical cell lines (Figure 3D,E). In HPV16 E6/E7‐immortalised cervical epithelial cells (Ect1/E6E7), VAMP7 overexpression was associated with an increase in apoptotic cell fractions, whereas in CC cell lines (SiHa, HeLa and C33A), VAMP7 overexpression was associated with relatively reduced apoptotic fractions. These effects exhibited opposite trends between immortalised epithelial cells and cancer cells. Considering the known crosstalk between autophagy and apoptosis, the observed differences in apoptotic profiles may reflect context‐dependent cellular responses accompanying VAMP7‐mediated changes in autophagy‐related processes.

**FIGURE 4:**
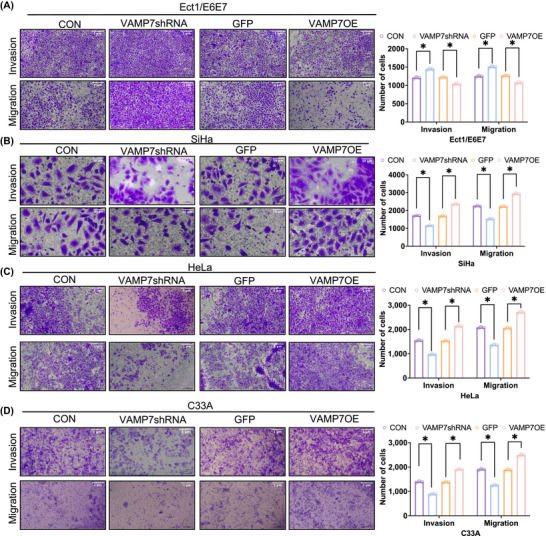
The effect of vesicle‐associated membrane protein 7 (VAMP7) regulation on invasion, and migration in cervical cells. (A) Representative images and quantification of Transwell invasion and migration assays in HPV16 E6/E7–immortalised cervical epithelial cells (Ect1/E6E7) following VAMP7 knockdown (VAMP7shRNA) or overexpression (VAMP7OE), with corresponding control groups (CON and GFP). (B) Representative images and quantification of Transwell invasion and migration assays in HPV16‐positive cervical cancer cells (SiHa) after VAMP7 knockdown or overexpression. (C) Representative images and quantification of Transwell invasion and migration assays in HPV18‐positive cervical cancer cells (HeLa) after VAMP7 knockdown or overexpression. (D) Representative images and quantification of Transwell invasion and migration assays in HPV‐negative cervical cancer cells (C33A) after VAMP7 knockdown or overexpression. Quantitative data are presented as mean ± standard deviation (SD) with individual data points from three independent experiments (*n* = 3). Statistical analysis was performed using two‐way analysis of variance (ANOVA). *p < .05. Scale bar = 1 µm.

To assess whether VAMP7 modulation is associated with changes in cell migratory and invasive behaviour, Transwell migration and invasion assays were performed. In HPV16 E6/E7‐immortalised cervical epithelial cells (Ect1/E6E7), VAMP7 overexpression was associated with reduced migratory and invasive capacity, whereas VAMP7 knockdown showed the opposite trend. In contrast, CC cell lines (SiHa, HeLa and C33A) exhibited increased migration and invasion upon VAMP7 overexpression and decreased motility following VAMP7 knockdown (Figure 4A–D). These findings suggest that VAMP7, through autophagy regulation, promotes invasion and migration in CC cells, while inhibiting these processes in non‐cancer cells.

**FIGURE 5:**
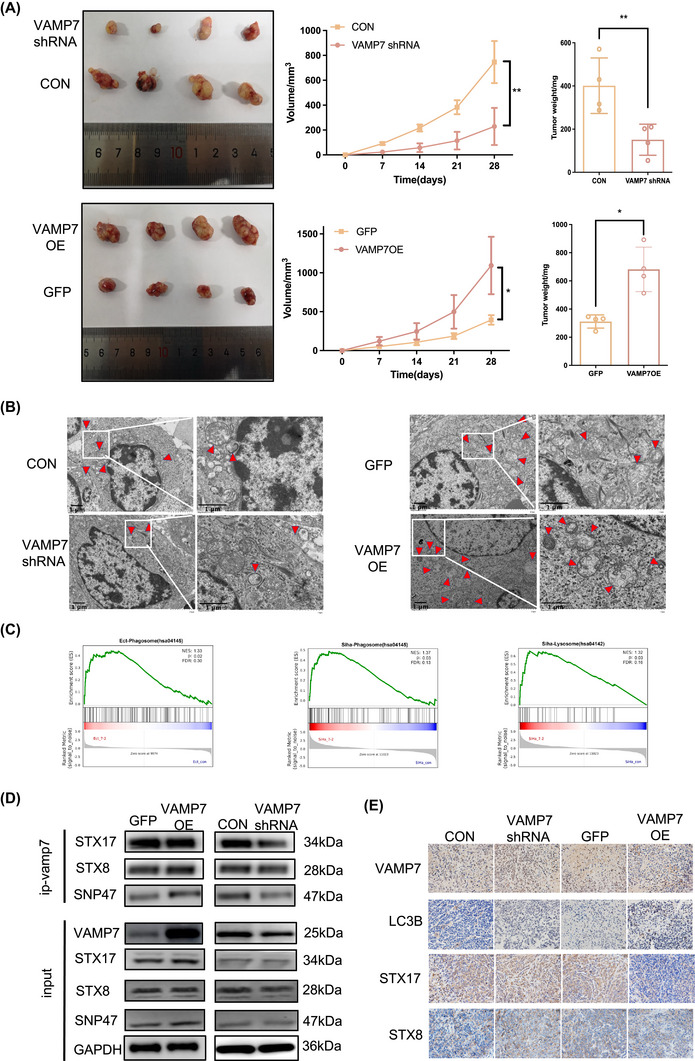
Mechanism of vesicle‐associated membrane protein 7 (VAMP7) in persistent HPV16 infection leading to cervical lesions. (A) Effects of VAMP7 regulation on tumour growth in nude mouse xenograft models. Tumour growth curves and final tumour weights are shown. Individual data points represent tumours from independent mice (*n* = 4 per group). (B) Representative transmission electron microscopy (TEM) images showing autophagosome formation in nude mouse xenograft models following VAMP7 regulation. Quantification of autophagic vesicles per cell is shown with individual data points (*n* = 4). Scale bar = 1 µm. Quantitative results are presented as mean ± standard deviation (SD) from independent samples (*n* = 4). Statistical significance was determined using two‐way analysis of variance (ANOVA). *p < .05. (C) Gene set enrichment analysis (GSEA) showing the significant pathways associated with VAMP7 modulation in HPV16‐infected cervical lesions. Key enriched pathways related to cellular processes regulated by VAMP7 are highlighted. (D) The effect of VAMP7 overexpression and knockdown on its interactions with soluble N‐ethylmaleimide‐sensitive factor attachment protein receptor (SNARE) complex‐related proteins STX7, STX18 and SNAP47 was assessed by co‐immunoprecipitation (Co‐IP). The interaction patterns and alterations in protein levels are presented. (E) Expression levels of SNARE signalling‐pathway‐related proteins regulated by VAMP7 in nude mouse tumour tissues. Western blotting analysis was performed to assess the protein expression changes upon VAMP7 modulation in tumour models.

To validate these results in vivo, we established a subcutaneous CC model in nude mice by injecting SiHa cells with stable VAMP7 overexpression or knockdown. VAMP7 knockdown significantly suppressed tumour growth, as evidenced by reduced tumour volume and weight compared to the control group. In contrast, VAMP7 overexpression significantly accelerated tumour growth, leading to larger and heavier tumours (Figure 5A). These results suggest that VAMP7 is a key regulator of tumour progression in CC.

Transmission electron microscopy of tumour tissues confirmed the in vivo autophagy regulation, showing fewer autophagosomes in the VAMP7 shRNA group and more autophagosomes in the VAMP7 overexpression group (Figure 5B). Additionally, IHC revealed decreased LC3B expression in the VAMP7 shRNA group and increased LC3B expression in the VAMP7 overexpression group (Figure 5E), further confirming that VAMP7 regulates autophagy during tumour progression.

Together, these results indicate that VAMP7 plays a pivotal role in regulating autophagy and modulating key phenotypic changes such as proliferation, apoptosis, migration and invasion in CC cells. VAMP7's differential effects on cancerous and non‐cancerous cells suggest that it may act as a modulator of autophagy in the tumour microenvironment, promoting cancer cell survival and invasion while inhibiting these processes in non‐malignant cells.

### 2.5 VAMP7 regulates autophagy in cervical lesions via SNARE complex formation

As previously discussed, VAMP7, a member of the SNARE protein family, plays a crucial role in autophagy regulation through its involvement in the fusion of autophagosomes with lysosomes.^10^, ^14^ Our earlier findings suggested that VAMP7 expression is downregulated during the early stages of HPV16 infection (from HPV‐negative to HPV16+) but progressively increases as lesions advance to HPV16+ HSIL and HPV16+ CA. Given the key role of VAMP7 in autophagy, we aimed to further investigate its function across different stages of cervical lesion progression, focusing on how its regulation might impact autophagic activity and disease progression.

**FIGURE 6:**
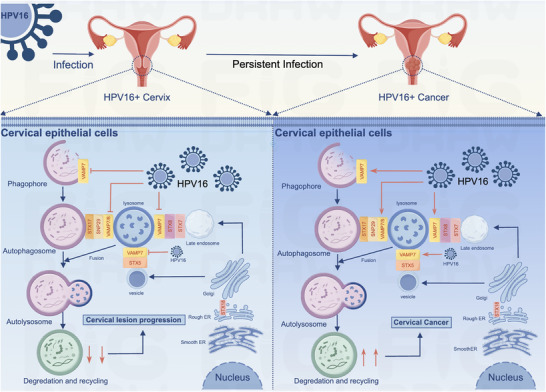
Mechanism of vesicle‐associated membrane protein 7 (VAMP7) in persistent HPV16 infection leading to cervical lesions. In the early stages of HPV16 infection, HPV16 promotes its persistent infection and the progression of cervical lesions by inhibiting VAMP7 and thereby autophagy. As the infection progresses, HPV16 enhances autophagy through VAMP7, but the lesions formed are not cleared, further promoting the development of cervical cancer. (Image by figdraw.)

To explore this, we selected HPV16 E6/E7‐immortalised cervical epithelial cells (Ect1/E6E7) and HPV16‐positive CC cells (SiHa) for VAMP7 overexpression and knockdown experiments. Our analysis involved RNA transcriptome sequencing to identify differentially expressed genes in both experimental setups, which provided insights into the mechanisms through which VAMP7 regulates autophagy in HPV16‐infected cells.

Gene set enrichment analysis (GSEA) enrichment analysis of the differentially expressed genes revealed significant pathways related to autophagosome–lysosome fusion, suggesting that VAMP7 modulates autophagic flux at this crucial step (Figure 5C). Additionally, the SNARE complex pathway, particularly involving VAMP7 and its interactions with Syntaxins (STX17, STX8 and SNP47), was also significantly enriched, supporting the hypothesis that VAMP7 mediates membrane fusion events critical for autophagy regulation.

To validate these findings, we conducted co‐immunoprecipitation (Co‐IP) experiments to investigate the interactions between VAMP7 and SNARE complex components (Figure 5D). The results confirmed that VAMP7 overexpression enhanced the binding affinity with Syntaxins, while VAMP7 knockdown reduced these interactions, demonstrating that VAMP7 directly influences SNARE complex assembly involved in autophagosome–lysosome fusion.

These findings provide compelling evidence that VAMP7 regulates autophagy in HPV16‐infected cervical epithelial cells through its modulation of SNARE complex formation. This mechanism contributes to the progression of cervical lesions by influencing autophagic activity, which is essential for both viral immune evasion and cancer development (Figure 6). Thus, VAMP7 emerges as a key mechanistic link in HPV‐driven cervical carcinogenesis and may represent a potential therapeutic target for intervention.

## 3 DISCUSSION

Persistent infection with high‐risk HPV is the primary cause of CC, one of the most common cancers in women worldwide.^16^ HPV16 and HPV18 are the most prominent high‐risk types, with HPV16 being the most virulent and responsible for a substantial proportion of cervical carcinogenesis.^4^ The progression from HPV infection to cervical lesions, including LSIL and HSIL, and eventually to CC, is a complex process driven by the viral oncogenes E6 and E7.^1^, ^17^, ^18^ These oncogenes interfere with critical cellular pathways, including cell‐cycle regulation,^19^, ^20^ apoptosis^21^ and DNA repair,^22^ leading to the accumulation of genetic mutations and promoting tumourigenesis.^4^

However, the role of host cellular processes in HPV‐driven carcinogenesis has gained increasing attention.^23^ Autophagy, a crucial cellular process for maintaining homeostasis by degrading damaged organelles and proteins, has emerged as a key player in this context.^7^, ^24^ Early‐stage autophagy in epithelial cells acts as a tumour‐suppressive mechanism, limiting oncogenic stress and preserving cellular integrity.^24^, ^25^, ^26^, ^27^ In contrast, during malignant transformation, cancer cells often hijack autophagy to adapt to metabolic stress, hypoxia and immune evasion, thereby facilitating tumour progression and metastasis.^25^, ^26^, ^27^, ^28^, ^29^, ^30^ In HPV‐associated malignancies, autophagy may play a dual role—protective in early stages but potentially pro‐tumourigenic in advanced disease.^9^, ^31^, ^32^, ^33^ Recent studies have also implicated vesicular trafficking and SNARE proteins, such as VAMP7, in regulating autophagic processes.^14^, ^15^, ^34^ SNARE proteins, which facilitate the fusion of autophagosomes with lysosomes, are essential for efficient autophagy.^10^, ^35^, ^36^ In this study, we identify VAMP7 as a key regulator of autophagy‐associated vesicular processes during the progression of HPV16‐related cervical lesions, specifically HPV16+ LSIL, HPV16+ HSIL and HPV16+ CA.

A significant observation in our study is the biphasic expression pattern of VAMP7 during cervical carcinogenesis. VAMP7 is downregulated in early‐stage HPV16‐positive lesions (HPV16+ LSIL), suggesting a potential tumour‐suppressive role early in the disease. However, VAMP7 expression increases significantly in advanced lesions (HPV16+ HSIL and HPV16+ CA), which aligns with the shift of autophagy from tumour suppression to tumour promotion as the disease progresses. This pattern mirrors the evolving role of autophagy itself, which is tumour‐suppressive in early lesions but pro‐tumourigenic in cancerous tissues.

Our findings further show that VAMP7 modulates various autophagy‐related features, including LC3 processing, autophagosome accumulation and SNARE protein expression. These findings underscore the importance of vesicular trafficking in autophagic flux, supporting a growing body of literature suggesting that SNARE‐mediated membrane fusion is a key step in regulating autophagy in HPV‐driven CC.^15^, ^36^, ^37^

Interestingly, VAMP7 is not only upregulated in HPV16‐positive CC but also in HPV18‐positive and HPV‐negative tumours, indicating that the role of VAMP7 in autophagy regulation extends beyond HPV16‐associated lesions. This finding suggests that VAMP7 upregulation may represent a more general feature of CC progression, irrespective of HPV genotype, and highlights the broader relevance of autophagy dysregulation in cervical carcinogenesis.

The observed biphasic expression and functional switch of VAMP7 during cervical lesion progression are consistent with current models in cancer biology that describe autophagy‐related pathways as transitioning from tumour‐suppressive roles in early stages to tumour‐promoting functions during malignant progression.^9^, ^31^, ^38^, ^39^, ^40^ As cervical lesions advance, increasing metabolic and invasive demands may promote the upregulation of VAMP7 to support tumour survival and metastasis.

In addition to experimental analyses, we examined the clinical relevance of VAMP7 expression in a retrospective cohort of CC patients. Elevated VAMP7 expression was associated with advanced FIGO stage, lymphovascular invasion and tumour recurrence. Although the limited sample size and retrospective design preclude definitive causal inference, these associations suggest a potential link between VAMP7 upregulation and aggressive clinicopathological features. Importantly, these clinical observations are concordant with our in vitro and in vivo findings, in which VAMP7 overexpression promoted tumour growth, invasion and autophagy‐associated phenotypes. Together, these data support the relevance of VAMP7 in CC progression at both experimental and clinical levels. Further validation in larger, independent and prospectively collected cohorts will be required to determine the prognostic significance of VAMP7 and to assess its potential clinical utility.

These results, together with our functional assays showing opposing effects of VAMP7 modulation in cancerous versus non‐cancerous cells, suggest that VAMP7 acts as a molecular amplifier of autophagy‐associated vesicular dynamics in CC. In non‐tumour cells, VAMP7 knockdown impaired autophagy and led to increased apoptosis, while in cancer cells, VAMP7 overexpression enhanced autophagosome formation, cell proliferation, migration and invasion, promoting tumour progression.

VAMP7 upregulation is not restricted to HPV16‐related cancers, supporting the idea that its role in autophagy regulation could be a shared feature of CC progression, rather than being specific to one HPV genotype. This broader relevance places VAMP7 at the intersection of HPV‐related and HPV‐independent mechanisms, linking autophagy, vesicular trafficking and tumour adaptation during cervical carcinogenesis.

In summary, our findings highlight the critical role of VAMP7 in regulating autophagy and vesicular trafficking during the progression of HPV‐driven cervical lesions. The biphasic expression pattern of VAMP7 suggests its dual role in CC progression—tumour‐suppressive in early disease and tumour‐promoting in advanced stages (Figure 6). Given its involvement in autophagy and tumour progression, VAMP7 may represent a potential therapeutic target for HPV‐related CC. Further studies are needed to fully elucidate the molecular mechanisms by which VAMP7 regulates autophagy and its interplay with the tumour microenvironment, providing valuable insights into potential therapeutic strategies for CC.

## 4 MATERIALS AND METHODS

### 4.1 Clinical sample collection

This study was approved by the Ethics Committee of the Obstetrics and Gynecology Hospital of Fudan University. Informed consent of all participants was obtained. The inclusion criteria were isolated samples from patients who underwent surgery in our hospital for cervical lesions or benign uterine diseases between 2014 and 2018. The samples were divided into five groups with 30 cases in each group: (1) control group (HPV negative and normal cervical tissue, HPV−), (2) HPV16+ group (HPV16 positive and normal cervical tissue, HPV16+), (3) HPV16+ LSIL group, (4) HPV16+ HSIL group and (5) HPV16+ CA group (HPV16 positive and CC tissue, CA). Immunohistochemical samples were collected from paraffin sections with complete clinical data and follow‐up data from our hospital during the same period, including 30 cases of HPV−, HPV16+, HPV16+ LSIL and HPV16+ HSIL cervical tissues and 68 cases of CC tissues, which included 30 of HPV16+CA, 22 of HPV18+CA and 16 of HPV‐CA. The exclusion criteria were patients who had received preoperative chemotherapy or radiotherapy. The diagnoses of all specimens were confirmed via histopathological examination. Cervical tissue samples included those for proteomic analysis and IHC. HPV infection status was determined by standardized polymerase chain reaction (PCR)‐based genotyping before sample collection. For HPV‐positive samples, HPV16 was the predominant genotype. HPV‐negative cervical tissues were used as biological controls, with no evidence of HPV DNA infection confirmed by clinical testing. Samples represented multiple pathological stages, including normal cervix, LSIL, HSIL and CC. Detailed clinical characteristics and HPV genotyping information for samples used in proteomics and IHC are summarised in Tables S1, S2, and S5.

### 4.2 Mass spectrometry data processing

After the proteins were extracted, we used LC–MS/MS to analyse the proteins. Mass spectrometric analysis was performed by using the EASY‐nLC 1000 (Thermo Scientific) liquid chromatograph and the Q Exactive HF mass spectrometer (Thermo Scientific). After the data acquisition, the raw files were analysed for protein identification using Maxquant v1.6.0.1 against the UniProt protein database with fixed carbamidomethyl modification of cysteine (C); variable oxidation of methionine (M); MS/MS tolerance 20 ppm. FDR < .01 was used as the cut‐off criterion.

### 4.3 Bioinformatics analysis of the proteins

Identified proteins were compared between HPV−, HPV16+, HPV16+ LSIL, and HPV16+ HSIL tissues, and HPV16+ cancer proteins with ≥ twofold change were considered to be differentially expressed proteins in cervical lesions. The differentially expressed proteins identified in cervical epithelia were analysed using the Protein Analysis Through Evolutionary Relationships (PANTHER) classification system, version 12 (http://www.panth erdb.org). The PANTHER program uses GO information to classify cell location, protein categories, molecular functions and biological processes. Proteomic data were also analysed using the ToppFun application within the ToppGene suite to identify enriched gene ontologies. All *p* values were obtained using the probability density function and Bonferroni correction was performed.

WGCNA^12^, ^13^ was used to construct the protein co‐expression network and find the module that was significantly related to the above groups. The network analysis was performed by the R package ‘igraph’,^14^ and four topological parameters, including degree, betweenness, closeness and clustering coefficient, were calculated to evaluate the importance of proteins in the identified module. GO enrichment analysis of biological process, molecular function and cell constituent were performed by using DAVID Bioinformatics Resources version 6.7. The protein–protein interaction (PPI) network was further processed by Cytoscape software. FDR < .05 was assigned as the threshold for significant enrichment.

### 4.4 Quantitative real‐time PCR (qRT‐PCR)

Total RNA was extracted using TRIzol reagent (Ambion). The Prime ScriptTM RT Reagent Kit was used to synthesise cDNA (Takara). Quantitative real‐time PCR was performed using the SYBR® Premix Ex TaqTM II PCR kit (Takara). All reactions were carried out in triplicate according to the manufacturer's instructions. β‐Actin was used as an endogenous housekeeping gene. The expression value was calculated by the comparative Ct method with the formula 2^−ΔΔCt^. All qRT‐PCRs were performed using the Applied Biosystems 7500 Real‐Time PCR system (Thermo Scientific). The primers used are listed in Table S3.

### 4.5 Western blot analysis

Twenty micrograms of protein were extracted from each sample, separated with 4%–20% SDS–PAGE gels, and then transferred to PVDF membranes. To prevent non‐specific binding, the membranes were blocked in 5% non‐fat dried milk at room temperature for 2 h. After blocking, the membranes were incubated overnight at 4°C with appropriately diluted primary antibodies (1:1000). Then, the secondary antibody (1:5000) was used for an additional 1 h incubation at room temperature. β‐Actin was used as Western blotting internal control. The membranes were visualised using the enhanced chemiluminescence (ECL) detection system (Bio‐Rad) and the Flurochem‐M imaging system (Protein simple). The bands were quantified using ImageJ software. Reagents are described in Table S4.

### 4.6 Immunohistochemistry

Paraffin sections were dewaxed, rehydrated and then underwent high‐temperature antigen repair. Subsequently, the sections were incubated overnight at 4°C with primary antibodies (1:50). Then, the secondary antibody (MaxVision kit, KIT‐5010) was used for 20 min of incubation at room temperature. After washing, the sections were counterstained with haematoxylin, dehydrated and sealed. The results were determined according to the histochemical score (H‐score) using Densito software in Quant Center. The percentage of immunostaining and the staining intensity were assessed in pixel by the identification of negative with the nuclei dyed blue, weak positive with colouring of light yellow, moderate positive with colouring of brown yellow and strong positive with colouring of dark brown. Reagents are described in Table S4.

### 4.7 Culture, transfection and construction of stable cells

The human cervical immortalised cell line Ect1/E6E7 (epithelial HPV16 E6/E7 transformed) was purchased from American Type Culture Collection. The human CC cell lines SiHa (HPV16‐positive cervical squamous cell carcinoma cell line), HeLa (HPV18‐positive cervical adenocarcinoma cell line) and C33A (HPV‐negative CC cell line) were purchased from The Cell Bank of Type Culture Collection of the Chinese Academy of Sciences. They were cultured in dulbecco's modified eagle medium (DMEM)/high glucose medium supplemented with 10% fetal bovine serum (FBS) (37°C, 5% CO_2_). Cells were cultured in 24‐well plates (1 × 10^5^ cells/well) for 18–24 h before transfection with lentiviral VAMP7 shRNA (the control group was the CON group) or VAMP7 overexpression plasmids (OE, the control group was the GFP group). Then, the virus‐containing medium was added in the presence of polybrene (6 µg/mL) for 4 h of incubation at 37°C. Seventy‐two hours after transfection, the cells were cultured in DMEM/high glucose medium containing puromycin (2 µg/mL) to construct stable transfected cells. Three different shRNA constructs targeting VAMP7 were purchased from Jikai Gene, and their sequences are shown in Table S6. All shRNAs were evaluated by qPCR and Western blotting, and VAMP7‐shRNA‐2, which produced the most efficient and reproducible knockdown, was selected for subsequent analyses (Figure S2A,B).

### 4.8 Cell proliferation assay

The cell proliferation assay was performed using the CCK8 assay (Dojindo Molecular Technologies, Inc.). A total of 1.0 × 10^4^ cells were seeded in 96‐well plates and cultured at 37°C for 6 h. Absorbance was measured at 450 nm using a microplate reader (Bio Rad). After 24, 48, and 72 h of incubation, the 450 nm absorbance of the solution was measured. The viability of the cells was calculated.

### 4.9 Transwell migration and invasion assays

Cell migration and invasion were evaluated by Transwell assays. For the cell invasion assay, the upper chamber was coated with Matrigel (BD Biosciences). Cells were resuspended in serum‐free DMEM and placed in the upper chamber (1 × 10^4^ cells/well). DMEM (1 mL) containing 10% FBS was added to the lower chamber. After 48 h, the cells invading the lower chamber were fixed in 4% paraformaldehyde and stained with crystal violet. Finally, the invasive cells were counted under a microscope (Olympus Corporation). For the cell migration assay, the experiment was carried out under the same conditions, except that the Matrigel was removed during the upper chamber precoating.

### 4.10 Cell‐cycle and apoptosis assays

Cell‐cycle analysis was performed by ethanol fixation. Cells were fixed with 70% ethanol overnight at 4°C, washed, and stained with 50 µg/mL propidium iodide (PI) and 50 µg/mL RNase for 15 min at room temperature away from light. Then, the cell cycle was detected by flow cytometry (FACS, Becton Dickinson). For the cell apoptosis assay, an Annexin V‐APC Apoptosis Detection kit (BD Biosciences) and flow cytometry were used according to the manufacturer's protocol.

### 4.11 Autophagy detected by transmission electron microscopy

Samples were first fixed with 2.5% glutaraldehyde at 4°C for at least 2 h to stabilise the cellular structures. Post‐fixation was carried out with 1% osmium tetroxide at room temperature for 1 h to further stabilise membrane structures. The samples were then dehydrated in a graded series of ethanol solutions (50% ethanol for 10 min, 70% ethanol for 10 min, 90% ethanol for 10 min, 95% ethanol for 10 min and 100% ethanol twice for 10 min each). Following dehydration, the samples were embedded in epoxy resin (Sigma Aldrich, 45345) through a sequence of immersions: a 1:1 mixture of epoxy resin and acetone for 2 h, a 3:1 mixture of epoxy resin and acetone for 2 h, pure epoxy resin overnight, and finally pure epoxy resin for an additional 4–6 h before polymerising at 60°C for 48 h. Ultrathin sections of 70–90 nm were then prepared and stained with 2% uranyl acetate for 20 min followed by lead citrate for 10 min to enhance contrast. The stained sections were observed and imaged using a transmission electron microscope (Philips, CM120), allowing for the detailed visualisation and analysis of autophagic structures.

### 4.12 Animal experiments

Female BALB/c (nu/nu) nude mice (4–6 weeks old) were purchased from Slack Laboratory Animal Co., Ltd. and raised in an specific pathogen‐free (SPF) environment. Each animal weighed approximately 18–20 g. To establish nude mouse models of CC to overexpress or interfere with VAMP7, each nude mouse was injected on one side of the subaxillary cortex with 5 × 10^6^ VAMP7‐overexpressing or disturbed SiHa cells. Tumour length and width were measured every 7 days using Vernier calipers, and tumour volume was calculated as follows: Volume = 1/2 × length × width^2^. The mice were humanely sacrificed at 28 days after implantation, and the xenograft tumours were collected for weighing and subsequent analysis. All animal experiments were approved by the Institutional Animal Care and Use Committee of Fudan University.

### 4.13 Statistical analysis

Data were analysed using SPSS 25.0 software. A non‐parametric test or one‐way analysis of variance (ANOVA) was used for comparisons between groups. Means of samples were compared by *t*‐tests or non‐parametric rank‐sum tests. The chi‐square test or Fisher test was used to analyse count data. Measurement data are expressed as the mean ± standard deviation (SD). Differences were considered statistically significant at *p* < .05.

## AUTHOR CONTRIBUTIONS

*Conceptualisation*: Weijuan Xin, Keqin Hua. *Methodology*: Weijuan Xin, Keqin Hua, Zhiling Zhu, Na Liu, Lu Zhou. *Investigation*: Weijuan Xin, Junjie Zhang, Na Liu, Lu Zhou. *Visualisation*: Weijuan Xin, Dan Wu, Xue Ding, Junjie Zhang. *Supervision*: Keqin Hua, Zhiling Zhu. *Writing – original draft*: Xue Ding, Dan Wu. *Writing – review & editing*: Dan Wu, Yue Wang, Weijuan Xin, Zhiling Zhu.

## CONFLICT OF INTEREST STATEMENT

The authors declare no conflicts of interest.

## ETHICS STATEMENT

This study was approved by the Ethics Committee of Obstetrics and Gynecology Hospital of Fudan University (Approval No. kyy2020‐166). All participants provided informed consent prior to sample collection. The study followed the ethical guidelines set by Obstetrics and Gynecology Hospital of Fudan University in accordance with the Declaration of Helsinki. Animal experiments were approved by the Institutional Animal Care and Use Committee (IACUC) at Fudan University (Approval No. kyy2020‐166), and all animal procedures were conducted in compliance with ethical standards.

## Supporting information



Supporting Information

Supporting Information

Supporting Information

Supporting Information

## Data Availability

All data are available in the main text or the supporting information.
