# Supplementary figures and images for: VAMP7‐mediated autophagy regulates cervical cancer progression associated with persistent HPV16 infection
Source: Clin Transl Med. 2026 Jan 8;16(1):e70590. doi: 10.1002/ctm2.70590 (PMC12783914; doi:10.1002/ctm2.70590)

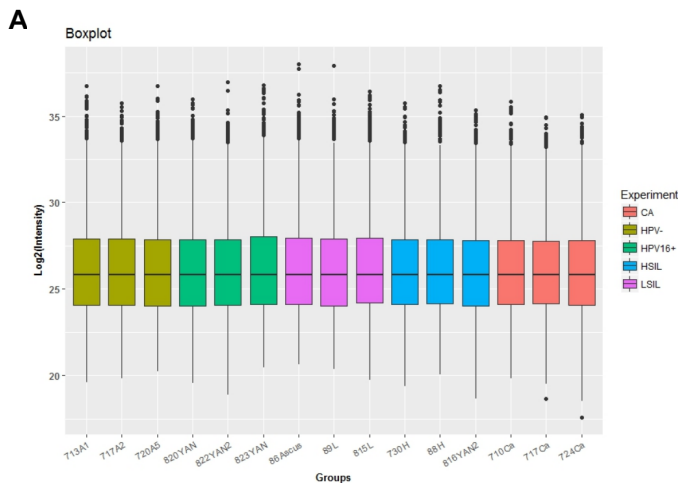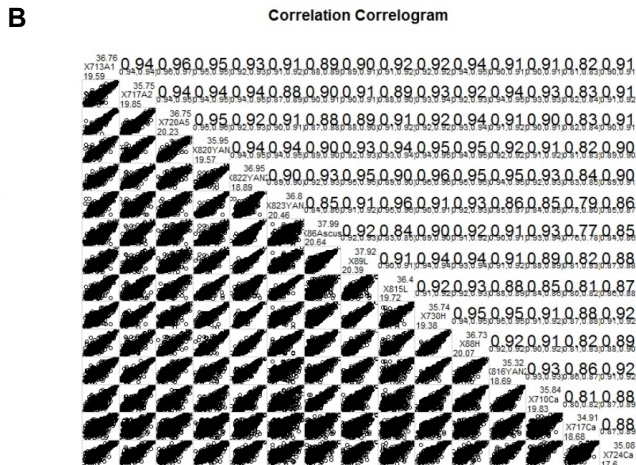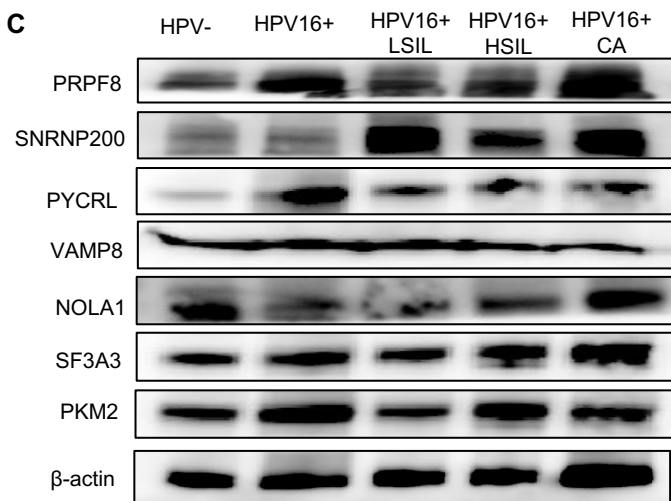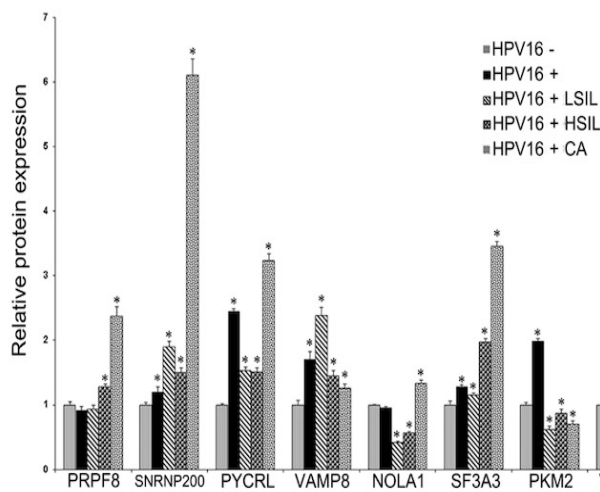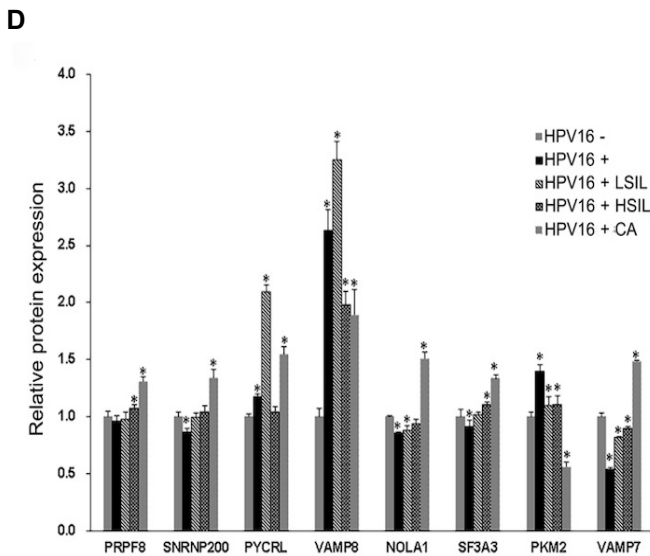

Supplement: Supplementary file 1 — Supporting Information [file CTM2-16-e70590-s002.pdf]

**A**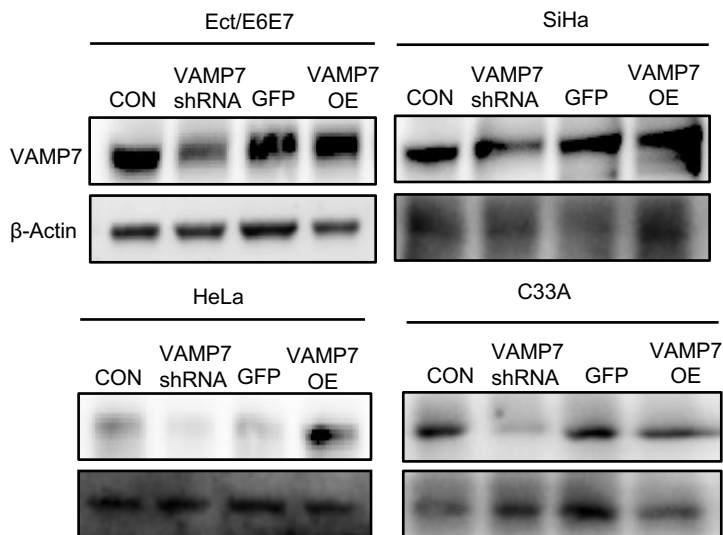**B**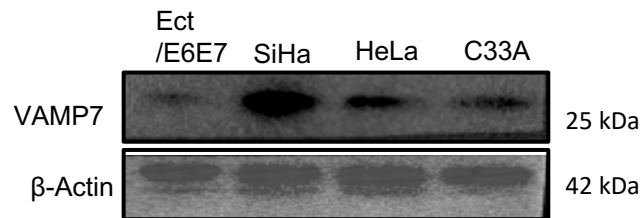**C**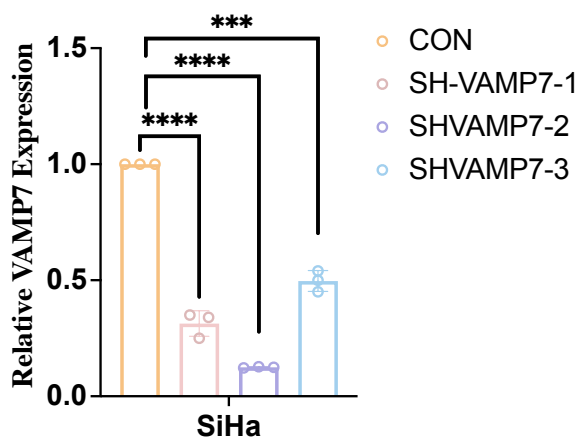

Supplement: Supplementary file 2 — Supporting Information [file CTM2-16-e70590-s004.pdf]
