# Supplementary material for: VAMP7‐mediated autophagy regulates cervical cancer progression associated with persistent HPV16 infection
Source: Clin Transl Med. 2026 Jan 8;16(1):e70590. doi: 10.1002/ctm2.70590 (PMC12783914; doi:10.1002/ctm2.70590)
Supplement: Supplementary file 4 — Supporting Information [file CTM2-16-e70590-s003.docx]

**Table S3 Gene primers for qRT-PCR**

| Gene | Primer | Sequences (5’-3’) |
| --- | --- | --- |
| VAMP8 | Forward | AAAATTCTGGTGGAAGAACGTG |
|  | Reverse | AAGAGCACAATGAAGAGGATGA |
| VAMP7 | Forward | CACTCTGAGAATAAGGGCCTAG |
|  | Reverse | GCTGAGCTACCAGATCTATGTT |
| β-actin | Forward | CCTGGCACCCAGCACAAT |
|  | Reverse | GGGCCGGACTCGTCATAC |

**Table S4 Reagent information**

| Reagent | Lot | Manufacturer |
| --- | --- | --- |
| Rabbit anti-human PRPF8 polyclonal antibody | #11171-1-AP,1:1000 | Proteintech, USA |
| Rabbit anti-human SNRNP200 polyclonal antibody | #23875-1-AP,1:1000 | Proteintech, USA |
| Mouse anti-human PYCRL monoclonal antibody | #TA502078,1:1000 | OriGene, USA |
| Rabbit anti-human VAMP8 polyclonal antibody | #GTX132181,1:1000/# CY6627,1:1000 | GeneTex, USA/Abways |
| Rabbit anti-human NOLA1 polyclonal antibody | #YN1713,1:1000 | Immunoway, USA |
| Rabbit anti-human SF3A3 polyclonal antibody | #YN1360,1:000 | Immunoway, USA |
| Rabbit anti-human PKM2 polyclonal antibody | #YT6197,1:1000 | Immunoway, USA |
| Rabbit anti-human VAMP7 polyclonal antibody | # YN2998,1:1000 | Immunoway, USA |
| Mouse anti-human β-actin monoclonal antibody | #66009-1-Ig,1:1000 | Proteintech, USA |
| HRP-conjugated sheep anti-rabbit and sheep anti-mouse secondary antibodies | #A0208, #A0216, 1:5000 | [Beyotime Biotechnology](https://www.baidu.com/link?url=WtNOI1ojokVsXT3LiWmCRpKb6oUFzLRgLiUe0nt4ZP44rdNDXhXftzvCK2Bfaqfv&wd=&eqid=94593386000b8e76000000046162d085), China |
| Rabbit anti-human STX7 polyclonal antibody | # YT7678,1:1000 | Immunoway, USA |
| Rabbit anti-human STX8 polyclonal antibody | # YT6429,1:1000 | Immunoway, USA |
| Rabbit anti-human STX17 polyclonal antibody | # YN4187,1:1000 | Immunoway, USA |
| Rabbit anti-human STX18 polyclonal antibody | # YN4330,1:1000 | Immunoway, USA |
| Rabbit anti-human LC3B polyclonal antibody | # CY5992,1:1000 | Abways, China |
| Mouse anti-human SNP47 polyclonal antibody | # sc-514428,1:500 | Santa Cruz Biotechnology,USA |
| Rabbit anti-human GAPDH polyclonal antibody | # 10494-1-AP,1:8000 | Proteintech, USA |
| MaxVision Kit( Rabbit/Mouse） | KIT-5010 | Maixin Biol, China |

**Table S5** **Clinical Characteristics of Patients**

|  | | Number of cases（%） | |
| --- | --- | --- | --- |
| Characteristics | Cases（N） | | Percent（%） |
| Total | | 68 | 100 |
| Age (years) | |  |  |
| <45 | | 28 | 41.18 |
| ≥45 | | 40 | 58.82 |
| Histological type  Squamous cell carcinoma  Adenocarcinoma  Adenosquamous carcinoma | | 46  10  8 | 67.65  14.71  11.76 |
| Small cell neuroendocrine carcinoma | | 4 | 5.88 |
| FIGO stages | |  |  |
| Ⅰ | | 30 | 44.12 |
| Ⅱ | | 15 | 22.06 |
| Ⅲ | | 23 | 33.82 |
| Histological grades | |  |  |
| high differentiation | | 25 | 36.76 |
| low/moderate differentiation | | 43 | 63.24 |
| Tumor diameters (cm) | |  |  |
| ≤2 | | 4 | 5.88 |
| >2,≤4 | | 43 | 63.24 |
| >4 | | 21 | 30.88 |
| Myometrial invasion | |  |  |
| Inner 1/3 | | 13 | 19.12 |
| Middle 1/3 | | 3 | 4.41 |
| Outer 1/3 | | 52 | 76.47 |
| Lymphovascular invasion | |  |  |
| Present | | 40 | 58.82 |
| Absent | | 28 | 41.18 |
| Aurgical margins | |  |  |
| Positive | | 3 | 4.41 |
| Negative | | 65 | 95.59 |
| Parametrial infiltration | |  |  |
| Present | | 14 | 20.59 |
| Absent | | 54 | 79.41 |
| Lower uterine segments involved | |  |  |
| Present | | 15 | 22.06 |
| Absent | | 53 | 77.94 |
| Lymph node metastasis | |  |  |
| Present | | 23 | 33.82 |
| Absent | | 45 | 66.18 |
| Other sites metastatic | |  |  |
| Present | | 3 | 4.41 |
| Absent | | 65 | 95.59 |
| Recrudesce | |  |  |
| Present | | 3 | 4.41 |
| Absent | | 65 | 95.59 |
| HPV typing | |  |  |
| 16+ | | 30 | 44.12 |
| 18+ | | 22 | 32.35 |
| Negative | | 16 | 23.53 |

**Table S6** **Oligonucleotide sequences used for VAMP7 shRNA construction**

| shRNA ID | Oligo | 5′ sequence | Target sequence (sense) | Loop | Target sequence (antisense) | 3′ sequence |
| --- | --- | --- | --- | --- | --- | --- |
| VAMP7-shRNA-1 | oligo-F | GATC | GCGAGTTCTCAAGTGTCTTAG | CTCGAG | CTAAGACACTTGAGAACTCGC | TTTTTT |
|  | oligo-R | AATTAAAAAA | GCGAGTTCTCAAGTGTCTTAG | CTCGAG | CTAAGACACTTGAGAACTCGC | — |
| VAMP7-shRNA-2 | oligo-F | GATC | GGAAAGAAGAAGTTACCATTA | CTCGAG | TAATGGTAACTTCTTCTTTCC | TTTTTT |
|  | oligo-R | AATTAAAAAA | GGAAAGAAGAAGTTACCATTA | CTCGAG | TAATGGTAACTTCTTCTTTCC | — |
| VAMP7-shRNA-3 | oligo-F | GATC | GCGAGGAGAAAGATTGGAATT | CTCGAG | AATTCCAATCTTTCTCCTCGC | TTTTTT |
|  | oligo-R | AATTAAAAAA | GCGAGGAGAAAGATTGGAATT | CTCGAG | AATTCCAATCTTTCTCCTCGC | — |
